# The Role of Diet in the Management of Chronic Idiopathic Urticaria: A Scoping Review

**DOI:** 10.7759/cureus.115321

**Published:** 2026-08-27

**Authors:** Olivia D'Alessio, Emma Huber, Jacob LeMaire, Berthmarie Camacho, Jeanette Rodriguez

**Affiliations:** 1 Family Medicine, Nova Southeastern University Dr. Kiran C. Patel College of Osteopathic Medicine, Clearwater, USA

**Keywords:** chronic idiopathic urticaria (ciu), chronic spontaneous urticaria (csu), diet(s), low histamine, pseudo-allergen free

## Abstract

Chronic idiopathic urticaria (CIU) is a dermatologic condition characterized by recurrent wheals, angioedema, or both for more than six weeks in the absence of an identifiable trigger. It is driven by spontaneous mast cell activation and the release of inflammatory mediators in the skin. Given its poorly understood pathogenesis, significant morbidity, and limitations of available treatment modalities, studies have suggested a link between diet and CIU. The primary objective of this study was to evaluate the literature published from 2015 to 2025 on this link, with an emphasis on dietary interventions, patient-reported outcomes, and gaps in the existing knowledge base. We conducted a scoping review following PRISMA guidelines, utilizing databases such as EMBASE, OVID Medline, and Web of Science. Search terms related to urticaria included “chronic idiopathic urticaria,” “chronic spontaneous urticaria,” “chronic idiopathic/spontaneous urticaria,” “CIU/CSU,” “CSU/CIU,” “spontaneous chronic urticaria,” and “idiopathic chronic urticaria,” combined with terms related to diet, including “diet,” “diet influence,” “diet regimen,” and “dietary effect.” The initial database search identified 121 records, of which nine met the eligibility criteria and eight were included after critical appraisal. The eight included studies examined the Mediterranean diet, a low-pseudoallergen diet, a pseudoallergen-free diet, a histamine-free, low-pseudoallergen diet, and a low-histamine diet, as well as micronutrient levels and inflammatory markers. The dietary interventions lasted from seven to slightly more than 39 days. The studies evaluated the effects of these diets on CIU disease activity, disease control, and quality of life using measures such as the Urticarial Activity Score, Urticarial Control Test, and Chronic Urticaria Quality of Life score. Outcomes tended to improve in the short term following the implementation of these diets, particularly in studies of diets limiting histamine and pseudoallergen intake. Additional findings included an association between CIU and a higher BMI. One study also reported higher vitamin D levels and lower vitamin B12 levels in patients with CIU than in the general population. The current body of literature is limited by small sample sizes, short intervention durations, and limited immunologic exploration. However, the consistency of patient-reported improvements across diverse geographic and dietary contexts underscores the potential role of diet as an adjunctive approach to CIU management. Future research should prioritize larger sample sizes, more balanced representation of the sexes, longer-term trials, and better control of participant-level confounding variables.

## Introduction and background

Chronic idiopathic urticaria (CIU), also referred to as chronic spontaneous urticaria (CSU), is characterized by the recurrent appearance of wheals, angioedema, or both for more than six weeks without an identifiable external trigger [1,2]. The condition is estimated to affect up to 1% of the general population and often causes significant morbidity, including impaired sleep, emotional distress, and reduced quality of life [2,3].

Epidemiology and clinical burden

CIU causes significant morbidity, although it is generally not associated with life-threatening complications. It affects quality of life through persistent pruritus, recurrent wheals, and angioedema, which disrupt sleep, impair concentration, and reduce work productivity [4]. Patients frequently report emotional distress, with elevated rates of anxiety and depression compared with the general population, highlighting the psychosocial burden of the disease [5]. Although CIU does not cause permanent organ damage, morbidity arises from its unpredictable course, frequent relapses, and prolonged duration in a subset of patients. The cumulative effect of these factors diminishes overall well-being; thus, research on this topic is vital.

Disease severity and treatment response in CIU are commonly assessed using validated patient-reported outcome measures. The Urticaria Activity Score over 7 days (UAS7) quantifies disease activity by measuring daily wheal count and itch severity; the Urticaria Control Test (UCT) evaluates overall disease control from the patient’s perspective; and the Chronic Urticaria Quality of Life Questionnaire (CU-Q2oL) assesses the impact of the disease on daily functioning and well-being. These instruments are widely used in clinical research to evaluate symptom burden and treatment effectiveness [6-8]. Despite extensive clinical evaluation, the etiology of CIU remains largely unclear in many patients, posing challenges for targeted management and prompting interest in adjunctive, nonpharmacologic strategies [3,9].

Current management strategies

Current management guidelines recommend a stepwise approach that begins with second-generation H1-antihistamines, progresses to higher doses when needed, and includes the addition of biologic therapy for refractory cases [10]. However, many patients experience suboptimal symptom control or express concerns about long-term medication use, driving the exploration of complementary strategies, including dietary modification [3,9,11].

Pathophysiology

The pathogenesis of CIU is believed to be primarily driven by spontaneous or inappropriate activation and degranulation of cutaneous mast cells, leading to the release of histamine and other inflammatory mediators. These mediators cause vasodilation and increased vascular permeability, resulting in plasma leakage into the skin and the formation of pruritic wheals and angioedema [12]. This inappropriate activation of cutaneous mast cells may involve two autoimmune endotypes: type I autoimmunity (autoallergy), mediated by IgE autoantibodies against autoantigens, and type IIb autoimmunity, mediated mainly by IgG autoantibodies against IgE or its high-affinity receptor, FcεRI [13]. There is also recognized heterogeneity in mast-cell-activating signals, including non-IgE-mediated pathways within a T-helper 2 (Th2) inflammatory milieu [14]. Emerging evidence suggests that diet may influence the course of CIU through various mechanisms, although this remains controversial [15]. Proposed mechanisms include the modulation of histamine release, pseudoallergic reactions to food additives, and the effects of specific dietary components on systemic inflammation [15].

Dietary mechanisms and histamine intolerance

One area of growing interest is the role of histamine intolerance, a condition in which impaired degradation of dietary histamine may contribute to symptoms such as flushing, headache, gastrointestinal discomfort, and urticaria. Diamine oxidase (DAO), an enzyme primarily expressed in the intestinal mucosa, plays a central role in the catabolism of exogenous histamine. In individuals with reduced DAO activity, whether due to genetic predisposition, intestinal inflammation, or dietary inhibitors, histamine accumulated from food sources may exacerbate or mimic urticarial symptoms. Although still under investigation, low DAO activity has been proposed as a contributing factor in a subset of patients with CIU. This has led to the use of low-histamine diets and DAO supplementation as potential therapeutic approaches [9,16,17].

In addition to low-histamine dietary interventions, pseudoallergen-free diets and elimination diets have been explored in both clinical and anecdotal contexts, but the evidence base remains fragmented and inconsistent [18-20]. A low-histamine diet is typically defined as one that avoids foods containing high levels of histamine (e.g., aged cheeses, fermented products, and processed meats) or foods that trigger histamine release (e.g., alcohol and biogenic amines), with the goal of reducing mast cell-mediated symptoms and cutaneous reactivity in patients with CIU [17]. In contrast, pseudoallergen-free diets aim to exclude naturally occurring and artificial non-immunologic triggers, such as food additives, preservatives, colorings, and salicylates, that are thought to exacerbate urticaria symptoms through non-IgE-mediated mechanisms [18,21,22]. Early investigations proposed that pseudoallergens may contribute to disease activity and reported substantial clinical improvement in selected patients following dietary elimination [18,22]. More recent prospective studies, however, suggest that although pseudoallergen-free diets may benefit a subset of patients, their effectiveness is more modest than initially reported [21]. Both dietary approaches overlap with broader elimination diets, in which patients systematically remove and reintroduce specific foods to identify potential triggers. However, there remains a lack of consensus regarding which dietary modifications, if any, are most effective or feasible in clinical practice [19,23].

Existing evidence and research gaps

Overall, the current body of literature examining diet in CIU is heterogeneous and fragmented across disciplines. Studies differ substantially in their dietary protocols, study designs, outcome measures, sample sizes, and diagnostic criteria, making direct comparisons difficult and limiting the ability to draw clinically meaningful conclusions. Although several narrative and systematic reviews have evaluated selected dietary interventions, the available primary evidence remains sparse, geographically diverse, and methodologically inconsistent [19].

A 2021 literature review graded dietary interventions based on the level of evidence supporting the effectiveness of each diet. It also emphasized the need for elimination diets to be maintained for at least 3 weeks to effectively measure outcomes [11]. However, such reviews are scarce, lack standardized criteria for study inclusion, and provide limited analysis of the statistical power of the included studies [19]. Comprehensive analyses of the quality and quantity of the existing literature remain limited. Furthermore, given the increased interest in integrative medicine in recent years and the continued controversy surrounding the role of diet in CIU management, a review focused on emerging research and research quality is warranted [24].

Rationale and objectives of the present review

Given the limited number of primary studies, the diversity of the dietary interventions investigated, and the variability in study methodology and outcome measures, a scoping review was selected as the most appropriate evidence-synthesis approach. Unlike systematic reviews, which are generally designed to address focused questions, and meta-analyses, which require sufficiently comparable studies, scoping reviews allow for the comprehensive mapping of an emerging body of literature, identification of research trends, and characterization of knowledge gaps without requiring methodological uniformity [25].

In conclusion, CIU causes significant morbidity and has a poorly understood pathophysiology, which complicates its management. Interest in dietary modification as an adjunctive strategy for dermatologic diseases continues to grow, although the overall role of nutrition in CIU remains controversial [15]. In particular, the evidence supporting pseudoallergen-free diets remains limited and inconsistent, despite reported benefits in selected patients [20]. Given the limited and methodologically diverse body of evidence currently available, a comprehensive synthesis of the recent literature is warranted. The objective of this scoping review is to map the existing evidence regarding diet and CIU, with particular emphasis on dietary interventions, patient-reported outcomes, and gaps in the current knowledge base. By identifying patterns within the literature and areas requiring further investigation, this review aims to inform future research directions and support evidence-based dietary recommendations for patients with CIU. It is important to note that some articles use the term chronic spontaneous urticaria, which is often used interchangeably with CIU. For consistency with the included literature, both terms will be used interchangeably throughout this paper.

## Review

Methods

Inclusion and Exclusion Criteria

To meet the inclusion criteria, articles were required to have been published on or after January 1, 2015. To maintain consistency throughout the review process, publication in English was predefined as an inclusion criterion. Abstracts, posters, and review studies were not accepted. Eligible studies included patients aged 17 years or older who had been diagnosed with CIU. No limitations were placed on ethnicity, race, sex, or culture. This broad approach was used to maximize the number of studies within the scope of the review and to focus on clinical outcomes related to diet and CIU. No specific diets were excluded, and the eligible studies examined dietary interventions, dietary patterns, or dietary nutrient levels. Scoping reviews, systematic reviews, other review articles, posters, and abstracts were excluded. Any study that could not be obtained in its entirety was also excluded. Animal studies were also excluded.

Information Sources

The search strategy was developed by a team consisting of the first and second authors. The initial search involved identifying key terms from relevant articles and searching the following databases: EMBASE, OVID MEDLINE, Web of Science, and PubMed. All searches were conducted on April 8, 2025, and the initial search strategy was adapted for each database.

Screening and Study Selection

The search identified 74 records in EMBASE, 14 records in OVID MEDLINE, 31 records in Web of Science, and two records in PubMed, for a total of 121 records. Tables 1-3 present the search protocol used for each database, along with the number of records identified.

**Table 1:** EMBASE search results.

Search No.	Search Strategy	Results
1	chronic idiopathic urticaria'/exp OR 'chronic spontaneous urticaria'	3,306
2	('chronic idiopathic/spontaneous urticaria' OR 'ciu/csu' OR 'csu/ciu' OR 'spontaneous chronic urticaria' OR 'idiopathic chronic urticaria'):ab,ti,kw	298
3	#1 OR #2	3,517
4	diet'/exp	468,925
5	diet' OR 'diet influence' OR 'diet regimen' OR 'dietary effect' OR 'diet*'	1,483,928
6	#4 OR #5	1,484,058
7	#3 AND #6	74

**Table 2:** Ovid MEDLINE® All search results.

Search No.	Search Strategy	Results
1	"chronic idiopathic urticaria".mp.	810
2	("chronic idiopathic urticaria" or "chronic idiopathic/spontaneous urticaria" or "ciu/csu" or "csu/ciu" or "spontaneous chronic urticaria" or "idiopathic chronic urticaria").mp.	904
3	1 or 2	904
4	Diet/	199,421
5	("diet" or "diet influence" or "diet regimen" or "dietary effect" or diet*).mp.	964,556
6	4 or 5	964,556
7	3 and 6	14

**Table 3:** Web of Science search results.

Search No.	Search Strategy	Results
1	TS=("chronic idiopathic urticaria" OR "chronic idiopathic/spontaneous urticaria" OR "ciu/csu" OR "csu/ciu" OR "spontaneous chronic urticaria" OR "idiopathic chronic urticaria")	2,184
2	TS=("diet" OR "diet influence" OR "diet regimen" OR "dietary effect" OR diet*)	661,469
3	#1 AND #2	31

After 14 duplicates were removed, 107 records remained for title and abstract screening, which was performed by all authors. During this stage, 20 records were excluded because they addressed the wrong topic, 24 because they were the wrong study type, five because they were published in an ineligible language, and 12 because they included the wrong population. Full-text retrieval and eligibility assessment were then conducted for 46 records. Each potentially eligible article was discussed among all reviewers, and all parties agreed on the final nine articles. Following full-text screening, 18 articles were excluded because they had the wrong study purpose, one because it was a missed duplicate, seven because they were not published within the specified period, three because they included the wrong population, and eight because the full text was unavailable. This resulted in the exclusion of 37 articles and the retention of nine articles. Following critical appraisal, eight articles were eligible for the final analysis. The selection and screening process was illustrated using a Preferred Reporting Items for Systematic Reviews and Meta-Analyses (PRISMA) flow diagram.

Search Strategy

The research question was formulated using the Population, Concept, and Context (PCC) framework. The Population was defined as individuals aged 17 years or older who had been diagnosed with CIU; the Concept was defined as clinical outcomes related to various diets used for symptom relief and management; and the Context was defined as studies published in English between January 1, 2015, and April 8, 2025. Based on these definitions, the guiding question was: ‘What is the impact of nutritional interventions on the management of CIU in individuals aged 17 years or older?’ Data collection took place on April 8, 2025, and searches were performed in the EMBASE, OVID MEDLINE, Web of Science, and PubMed databases. These databases were selected because they contain a large number of primary health research articles relevant to this review.

EMBASE is a comprehensive database covering published literature in the biomedical and pharmacological sciences [26]. MEDLINE, maintained by the National Library of Medicine, is the primary component of the publicly accessible PubMed database [27]. Web of Science provides access to a wide range of medical and scientific literature for review and analysis [28].

The first and second authors, both medical students, independently conducted the searches using search terms that included Medical Subject Headings (MeSH) terms and keywords such as “chronic idiopathic urticaria,” “chronic spontaneous urticaria,” and “diet(s).”

This review included studies examining clinical outcomes among patients with CIU following various nutritional plans, including the Mediterranean diet, low-pseudoallergen diet, and low-histamine diet. Studies that measured dietary micronutrient levels, current dietary habits, or CIU disease activity were also included. The exclusion criteria included articles published before 2015, studies published in languages other than English, studies involving patients younger than 17 years, abstracts, poster presentations, and review studies. Experimental and quasi-experimental studies were included, along with analytical and descriptive observational studies. The PRISMA reporting framework was used to organize and standardize the study-selection process [29].

Selection of Sources of Evidence

To ensure consistency in the screening process, all reviewers initially assessed the same set of 46 records selected for full-text review and discussed their results, along with any necessary adjustments to the screening and data-extraction protocols. During the formal selection process, four reviewers worked in pairs to assess the titles, abstracts, and available full texts of all potentially eligible publications. Any disagreements regarding study selection were resolved through discussion and consensus, with additional reviewers consulted as needed.

Data Charting Process

Data were extracted and charted from the articles by two or more independent reviewers. Rayyan AI was used to facilitate blinded screening, apply the inclusion and exclusion criteria, and streamline collaboration during the study-selection process. The draft data-extraction tool was modified and revised as necessary by the reviewers during the extraction of data from each included source. Any disagreements regarding study selection or data extraction were resolved through discussion and consensus, with additional reviewers consulted as needed. The Rayyan project was updated sequentially throughout the process. At the end of the screening process, nine articles remained. Because of the diversity of the study designs, dietary interventions, outcome measures, and statistical reporting among the included studies, the findings were synthesized narratively rather than through a quantitative meta-analysis.

Data Items

The summary tables included the article title, authors, year of publication, and country of origin. Data were summarized according to the aims or purposes of the articles, study populations and sample sizes, methodologies used, and study designs (e.g., cross-sectional, case-control, or interventional cohort studies). The main findings and limitations of the studies were also included.

Quality Assessment

The nine articles retained following Tier II data extraction were evaluated using the Joanna Briggs Institute (JBI) Critical Appraisal Tools [30]. These tools assist in assessing the methodological quality of different study types, including case-control studies, cohort studies, and randomized controlled trials. They help identify potential methodological limitations and risks of bias and support the evaluation of the trustworthiness of the articles included in this scoping review.

Using the appropriate checklist for each study design, two researchers independently scored the nine candidate studies, considering potential sources of bias, methodological congruity, and the relevant checklist domains. The appraisal results were used to classify the articles as having a high, moderate, or low risk of bias. Each article was assessed using the JBI checklist corresponding to its study design to evaluate its methodological quality. The two researchers compared their appraisal scores, discussed any differences, and reached a consensus regarding which articles should be included. Studies demonstrating a low-to-moderate risk of bias based on the applicable JBI checklist criteria and reviewer consensus were considered eligible for final inclusion in this scoping review. Eight of the nine candidate studies were included; one article was excluded because it was too brief and lacked sufficient evidence, discussion, and references.

Figure 1 shows the PRISMA flow diagram of the study-selection process.

**Figure 1:**
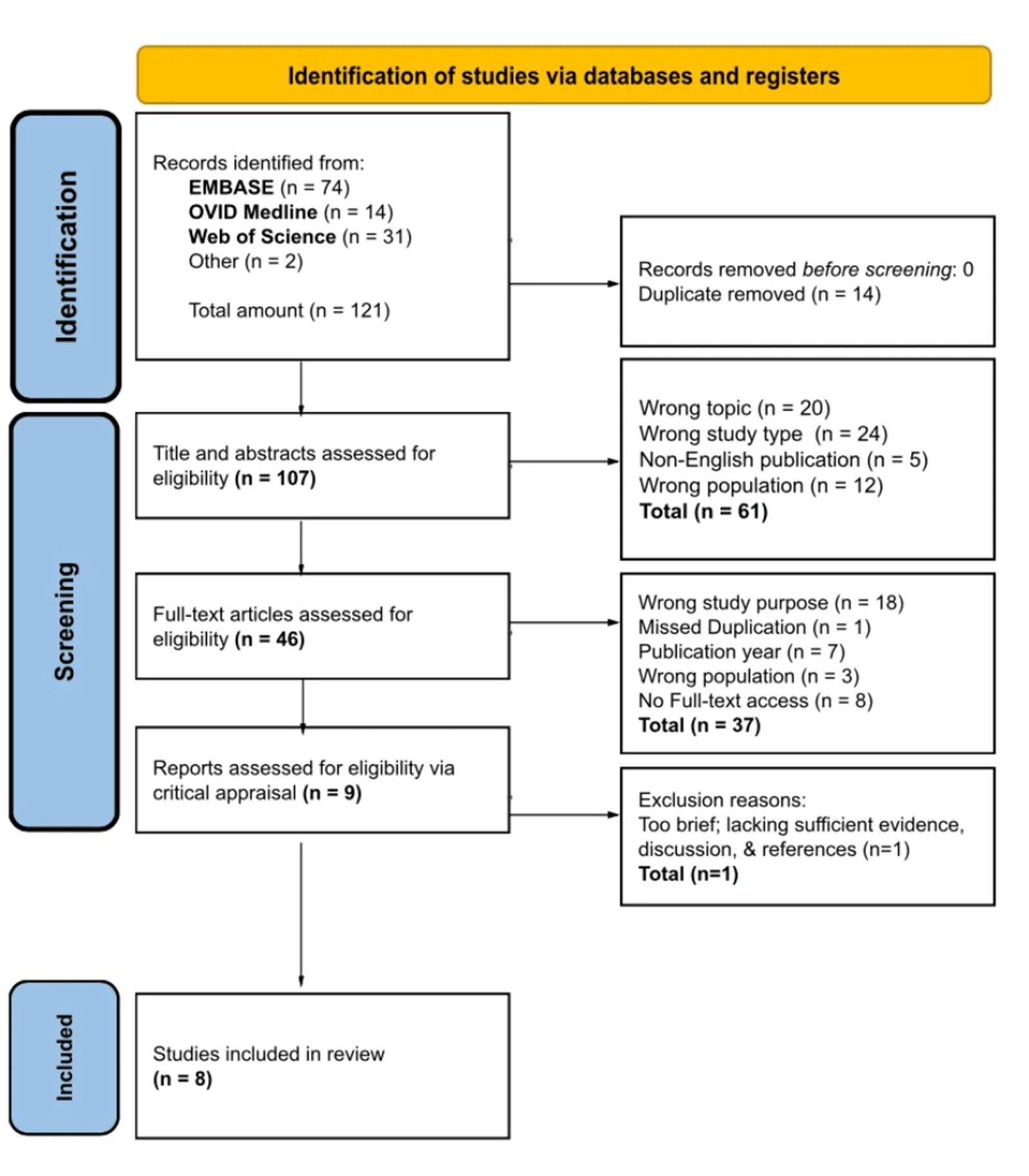
PRISMA flow diagram of study selection. PRISMA: Preferred Reporting Items for Systematic Reviews and Meta-Analyses.

Results

A summary of the findings from each article included in the final analysis is presented in Table 4.

**Table 4:** Summary table of studies included in the final review. ASST: Autologous Serum Skin Test; BDHQ: Brief-type Self-administered Diet History Questionnaire; CSU: Chronic Spontaneous Urticaria; CU: Chronic Urticaria; CU-Q₂oL: Chronic Urticaria Quality of Life Questionnaire; DAO: Diamine Oxidase; DLQI: Dermatology Life Quality Index; IFN-γ: Interferon Gamma; IL-6: Interleukin 6; MCHC: Mean Corpuscular Haemoglobin Concentration; MCV: Mean Corpuscular Volume; MDS: Mediterranean Diet Score; PAFD: Pseudoallergen-Free Diet; UAS: Urticaria Activity Score; UAS4: Urticaria Activity Score over 4 days; UAS7: Urticaria Activity Score over 7 days; UCT: Urticaria Control Test; USS: Urticaria Severity Score.

Author and Year	Aim of Study	Country	Study Design	Study Population and Setting	Methods	Main Findings	Limitations	Authors’ Conclusions
Ayvaz HH ands Kuyumcu A (2021) [6]	To investigate the relationship between adherence to the Mediterranean diet and chronic spontaneous urticaria (CSU).	Turkey	Cross-sectional case-control observational study	100 patients with CSU (30 men and 70 women; mean age, 38.6 ± 13.0 years) and 100 age- and sex-matched healthy controls (30 men and 70 women; mean age, 38.7 ± 13.8 years).	Adherence to the Mediterranean diet was assessed using a validated 14-item questionnaire. Urticaria severity and control were assessed using the Urticaria Activity Score over 7 days (UAS7) and Urticaria Control Test (UCT), respectively.	The mean UAS7 score among patients with CSU was 23.73 ± 11.34, and the mean UCT score was 8.66 ± 3.60. UAS7 was negatively correlated with the Mediterranean Diet Score (MDS), whereas UCT was positively correlated with MDS.	The study did not consider psychological stress or physical activity levels.	Adherence to the Mediterranean diet may be independently associated with a reduced likelihood and severity of CSU.
Maheshwari A et al. (2022) [31]	To explore etiological factors associated with CSU and evaluate interleukin 6 (IL-6) and interferon gamma (IFN-γ) levels.	India	Case-control study	60 adults with CSU (mean age, 33.18 ± 9.59 years; 73.33% female) and 60 age- and sex-matched healthy controls (mean age, 30.7 ± 8.3 years).	Participants underwent biochemical and hematological investigations, hepatitis serology, thyroid function testing, antithyroid antibody testing, IL-6 and IFN-γ measurement, and the autologous serum skin test (ASST). UAS7 was calculated for all patients and repeated after avoidance or treatment of an identified etiological factor.	IFN-γ levels were significantly higher among patients with CSU and among those with higher UAS7 scores. Of the four patients who reported food intolerance, only one improved following a low-pseudoallergen diet.	Small sample size; food and drug provocation tests were not performed; and skin-prick testing, serum immunoglobulin E measurement, and the basophil histamine-release assay were not performed.	The authors concluded that elevated IL-6 and IFN-γ levels suggest the involvement of both T-helper 1 and T-helper 2 immune responses in CSU. Antithyroid antibodies, ASST, and patch testing may be considered during the evaluation of patients with CSU.
Matano Y et al. (2020) [7]	To evaluate dietary habits and nutrient intake and their associations with CSU.	Japan	Case-control study	70 adults with CSU (29 men and 41 women) recruited from outpatient dermatology clinics and 70 age- and sex-matched healthy hospital volunteers and employees.	Participants completed the Brief-type Self-administered Diet History Questionnaire (BDHQ), and disease control was assessed using the UCT. Multiple logistic regression was used to assess the variables.	CSU was associated with higher egg intake (odds ratio, 1.05) and higher body mass index (BMI). Uncontrolled CSU (UCT ≤11) was associated with higher beverage intake (odds ratio, 1.01). Coffee and caffeine-rich and nonalcoholic beverage consumption was also higher among patients with uncontrolled CSU.	The study was conducted at a limited number of locations in Japan, and dietary habits may differ across regions. Baseline disease activity was not assessed, only one time point was evaluated, and the BDHQ did not account for micronutrient supplementation.	Although weak associations were observed between eggs, BMI, and CSU and between beverage intake and uncontrolled CSU, further research is needed to determine whether these factors influence the development or progression of CSU.
Sarac E et al. (2022) [32]	To investigate the effects of a pseudoallergen-free diet on disease severity, antihistamine use, and quality of life in patients with CSU.	Turkey	Prospective single-center, single-group pilot study	23 patients (19 women and 4 men; mean age, 43 ± 4.6 years; range, 18–67 years) with poorly controlled CSU lasting at least 6 months.	Patients recorded their daily Urticaria Activity Score (UAS) during a baseline week and then followed a pseudoallergen-free diet (PAFD) for 3 weeks. UAS, Chronic Urticaria Quality of Life Questionnaire (CU-Q₂oL) scores, and antihistamine use were assessed.	Six patients (26.1%) had a strong response (ΔUAS ≥8), six (26.1%) had a partial response (8 > ΔUAS ≥4), and 11 (47.8%) had no response (ΔUAS <4). Nine patients (39.1%) had significantly improved quality of life, and six (26.1%) experienced both reduced disease activity and improved quality of life. Antihistamine use decreased in 43.5% of patients.	Small sample size; lack of a control group and provocation testing; no restrictions on nonsteroidal anti-inflammatory drugs or aspirin use; antihistamine use was not fully restricted; and clinical or laboratory predictors of response were not assessed.	A PAFD may reduce disease activity and antihistamine use and improve quality of life in selected patients with CSU when used alongside conventional treatment.
Siebenhaar F et al. (2016) [33]	To determine the frequency of histamine intolerance among patients with CSU and its relevance as a potential underlying cause.	Germany	Prospective interventional cohort study	157 patients with moderate-to-severe CSU (118 women and 39 men; median age, 43 years; range, 17–79 years) and a median disease duration of 2 years.	Participants provided clinical histories concerning reactions to histamine-rich foods. They then followed a histamine-free, low-pseudoallergen diet for 31 days, followed by a double-blind, placebo-controlled 75-mg oral histamine challenge. Symptoms were monitored for 48 hours after the challenge.	A history of histamine intolerance was reported by 34% of patients, but mean UAS7 scores did not differ significantly between those with and without such a history. Following the diet, 46% had a reduction of at least 7 points in UAS7. Oral histamine provocation produced a positive wheal response in 17%. There was little association among patient history, dietary response, and provocation results, and only 2 of 157 patients were positive in all three domains.	There was no control group following a normal diet, and the dietary phase was not blinded; therefore, a placebo effect could not be excluded.	Avoidance diets low in pseudoallergens may improve urticaria symptoms, but the improvement is probably not attributable to the absence of dietary histamine.
Son JH et al. (2018) [34]	To evaluate the role of ingested histamine and investigate the effects of a histamine-free diet in adults with chronic urticaria (CU).	Korea	Prospective single-group interventional study	22 patients (11 women and 11 men; mean age, 41.2 ± 16.1 years; range, 18–70 years). The mean duration of treatment before enrolment was 22 months.	Participants followed a histamine-free diet for 4 weeks. Urticaria severity was assessed using the Urticaria Severity Score (USS) and UAS7. Plasma histamine levels and diamine oxidase (DAO) activity were measured before and after the diet.	After 4 weeks, the mean USS decreased from 25.0 ± 11.4 to 16.2 ± 12.8 (p = 0.010), and UAS7 decreased from 10.3 ± 9.2 to 4.1 ± 4.1 (p = 0.006). Plasma histamine levels also decreased significantly (p = 0.010), whereas DAO activity did not change significantly (p = 0.165). Antihistamines were continued during the study.	Long-term antihistamine use may have influenced DAO activity, and medication duration and dosage varied among patients. The dietary intervention was brief, the sample size was small, and developing individualized histamine-free menus required assistance from the Department of Nutrition.	Ingested histamine may be associated with CU severity, and a histamine-free diet may be a helpful adjunctive treatment for adults with CU.
Wagner N et al. (2017) [8]	To evaluate the effects of a low-histamine diet on symptoms and quality of life among patients with CSU.	Germany	Prospective single-group interventional study	56 patients with CSU and gastrointestinal symptoms (42 women and 14 men) recruited from two specialized urticaria centres.	Participants followed a low-histamine diet for at least 3 weeks. UAS was recorded daily, and quality of life was assessed at screening, baseline, and after the diet using the Dermatology Life Quality Index (DLQI) and CU-Q₂oL. DAO activity was measured before and after the diet.	Overall, 75% of patients benefited from the diet. Thirty-four of 56 patients (61%) achieved the primary endpoint of an improvement of at least 3 points in UAS4. Mean UAS4 decreased from 9.05 to 4.23 (p = 0.004), and the mean reduction among severely affected patients was 8.59 points (p < 0.001). DAO activity remained unchanged.	The absence of blinding and a control group may have introduced bias through a placebo effect.	A low-histamine diet may reduce symptoms and improve quality of life among patients with CSU and gastrointestinal symptoms.
Wu CH et al. (2015) [35]	To explore the association between micronutrient levels and CSU.	United Kingdom	Retrospective database analysis with comparison to population survey data	282 patients aged 19–64 years with CSU identified from a pre-existing database at Southampton General Hospital; most patients were female.	Vitamin D, vitamin B12, ferritin, mean corpuscular volume (MCV), and mean corpuscular haemoglobin concentration (MCHC) levels were extracted from the hospital database and compared with age-matched data from the United Kingdom National Diet and Nutrition Survey.	Patients with CSU had higher vitamin D levels than the general United Kingdom population (51.4 ± 27.03 vs. 45.4 ± 24.84 nmol/L; p = 0.001), although 54.6% had levels below 50 nmol/L. Vitamin B12 levels were significantly lower (284 ± 108.4 vs. 359 ± 131.2 ng/L; p < 0.001). Ferritin levels were significantly lower among men with CSU (p = 0.009) but not among women (p = 0.096). Overall, 4.5% had low vitamin B12 levels, and 8.5% had low ferritin levels. MCV was not a reliable indicator of vitamin B12 or iron deficiency.	Dietary and medication histories were not recorded; therefore, differences in diet or supplementation may have contributed to the observed micronutrient differences.	It remains unclear whether the low-normal vitamin B12 and ferritin levels observed among patients with CSU reflect inadequate dietary intake or an association with the disease. The effects of supplementation remain unknown.

Characteristics of Studies

The eight included studies were conducted in six different countries, none of which were in the United States. Germany and Turkey each contributed two studies, whereas Korea, Japan, India, and the United Kingdom contributed one study each [6-8,31-35].

Four of the eight studies had sample sizes greater than 100 participants, ranging from two matched groups of 70 participants each to 282 participants [6,7,33,35]. Of the remaining four studies, one included 22 participants [34], another included 23 participants [32], another included 56 participants [8], and the fourth included 60 participants [31]. Most study participants were female, except in one study in which the patient and control groups each included 70 male and 30 female participants [6] and another study that included equal numbers of male and female participants [34]. The mean age of participants across the studies ranged from 33.18 to 43 years.

The majority of the study designs used to evaluate the role of diet in CIU management were case-control studies, with five of the eight studies using this design [6,7,31,32,35]. Among these, one was a cross-sectional case-control study [6], and another was an observational case-control study [35]. The remaining studies were a prospective interventional cohort study [33], a prospective cohort study [34], and a prospective single-group study [8].

The duration of each study was typically brief, ranging from 7 to 39 days [6,8,31-34]. The typical model involved participants following the diet of interest for a minimum of 3 weeks, with urticarial activity measured after the designated number of weeks. All studies that followed this model used the weekly Urticaria Activity Score (UAS7) to measure disease activity and the severity of CIU symptoms [6,8,31-34]. Two studies did not follow participants over time but instead used questionnaires and data analysis [7,35]. These were also the only studies that did not use UAS7. Two studies used the Urticaria Control Test (UCT) to measure CIU control [6,7]. Two studies used the Chronic Urticaria Quality of Life Questionnaire (CU-Q2oL) to evaluate participants’ quality of life [8,32].

Key Findings

The eight studies evaluated the roles of various diets and diet-related factors and their associations with CIU/CSU. These included the Mediterranean diet [6], a pseudoallergen-free diet [32], a histamine-free, low-pseudoallergen diet [33], a low-histamine diet [8,34], micronutrient levels [35], specific dietary factors [7], and inflammatory markers [31].

Ayvaz et al. concluded that UAS7 scores were negatively correlated with Mediterranean Diet Scores (MDSs), whereas UCT scores were positively correlated with MDSs, suggesting better disease control among patients with greater adherence to the Mediterranean diet [6]. Maheshwari et al. found that interleukin-6 (IL-6) and interferon-gamma (IFN-γ) levels were significantly elevated in patients with CIU compared with age-matched controls. IFN-γ levels were also significantly elevated among patients with higher UAS7 scores [31]. Sarac et al. found that 26.1% of patients demonstrated a strong response to the pseudoallergen-free diet (ΔUAS ≥ 8), 26.1% demonstrated a partial response (4 ≤ ΔUAS < 8), and antihistamine use decreased in 43.5% of participants [32]. Siebenhaar et al. found that 46% of patients with CSU experienced reduced disease activity after following a histamine-free, low-pseudoallergen diet for 1 month [33].

Son et al. used the Urticaria Severity Score (USS) and UAS to measure symptom severity before and after a low-histamine diet. They demonstrated significant reductions in urticaria severity following a histamine-free diet, with UAS scores decreasing from 10.3 ± 9.2 to 4.1 ± 4.1 after 4 weeks of intervention (p = 0.006) [34]. Wagner et al. found that 75% of patients benefited from following a low-histamine diet for 3 weeks, with UAS4 scores decreasing significantly from 9.05 to 4.23 [8]. The Dermatology Life Quality Index (DLQI) and CU-Q2oL were used to assess quality of life, and both indicated improved quality of life among patients with CSU after the dietary intervention [8].

Using the Brief Diet History Questionnaire (BDHQ), one study found that patients with CSU had a higher intake of eggs than age-matched controls. The UCT was also used to assess disease control, demonstrating that patients with better disease control had a lower intake of coffee. Patients with CSU also had a higher mean BMI than controls: 23.8 kg/m² compared with 20.9 kg/m², respectively [7]. Wu et al. found that micronutrient levels were associated with CSU. Patients with CSU had significantly lower vitamin B12 levels than controls (p < 0.001), whereas ferritin levels were significantly lower among male patients with CSU than among male controls (p = 0.009) [35].

Limitations

Several studies had sample sizes of fewer than 100 participants [8,31,32,34], which may have reduced their statistical power and resulted in some findings not reaching statistical significance. However, only statistically significant findings are reported in this scoping review. One study did not account for participants’ physical activity or psychological stress [6]. Some studies did not account for whether participants were taking micronutrient supplements in addition to following their diets [7,35]. Medication use, including nonsteroidal anti-inflammatory drugs (NSAIDs) and aspirin, was also not fully controlled in some studies [32,35].

Gaps in Research

None of the studies were conducted in the United States; therefore, the generalizability of the findings beyond populations in Korea, Japan, India, Turkey, Germany, and the United Kingdom may be limited. Additionally, most dietary intervention studies assessed adherence for only 3-4 weeks; therefore, outcomes beyond this period remain unknown.

Discussion

The evidence presented in this review maps the current literature published from January 2015 to April 2025 on the relationship between dietary interventions and CIU. Based on both the quality and quantity of the existing literature, these findings provide insight into patient-reported outcomes, potential nutritional strategies, and significant gaps in the knowledge base. Although only eight studies met the final inclusion criteria, this finding likely reflects the limited availability of recent, methodologically rigorous primary studies evaluating dietary interventions in CIU. The predefined eligibility criteria were intentionally designed to prioritize study quality and clinical relevance, thereby providing a more reliable synthesis of the available evidence. Although preliminary evidence suggests that dietary modification may improve symptoms and quality of life, the literature remains limited overall in scope, design quality, and mechanistic depth. Together, these findings outline a roadmap for future research into the nutritional management of CIU.

Study Design

The majority of the interventional studies followed a simple structure: measurement of baseline urticaria activity, implementation of a dietary intervention, and reassessment after 1 month. Only one of the eight studies was a retrospective database review; most were prospective case-control studies. This design introduces vulnerability to the placebo effect, particularly given the short intervention periods (<2 months). If the mechanism of benefit involves lowering the inflammatory threshold, the current study durations may be insufficient to capture a true response curve or plateau of effect. Longer trials are therefore essential.

Most studies focused on disease activity and quality-of-life outcomes. A smaller subset also examined histamine intolerance, micronutrient deficiencies, or CIU pathophysiology. However, because the predominant outcome measures were symptom- and quality-of-life-based, the mechanistic role of diet in CIU pathogenesis remains difficult to establish. Notably, only Siebenhaar et al. performed provocation testing, which demonstrated that pseudoallergen avoidance improved symptoms independently of dietary histamine. Future studies should incorporate provocation testing to more precisely identify dietary triggers and the recurrence of symptoms upon re-exposure.

Study Demographics

Study demographics were markedly skewed toward female participants. Only one study enrolled a majority of male participants [6], and only one had an equal sex distribution [34]. This imbalance reflects the higher prevalence of CIU among women, who are affected approximately twice as often as men. Nevertheless, several studies reported female representation exceeding that of men by three- to five-fold, raising the possibility that sex-specific nutritional needs, hormonal influences on metabolism, or immune differences could have influenced the outcomes. Future studies should control for sex as a biological variable to clarify its role in CIU and dietary response. Additionally, the minimum eligible age for this review was 17 years; Siebenhaar et al. included participants aged 17-79 years [33].

The seven studies were geographically diverse, spanning East Asia, South Asia, Western Asia, and Western Europe. This geographic distribution may reduce the likelihood that the findings are attributable to regional diet or microbiome alone and suggests that certain dietary triggers may transcend cultural contexts. Importantly, this geographic representation also indicates that effective dietary strategies need not require abandoning culturally ingrained food practices.

Quality of life and disease activity were central endpoints in most studies and were typically measured using the Chronic Urticaria Quality of Life Questionnaire and self-reported scales such as the Urticaria Activity Score, respectively. Although dietary interventions generally improved patient-reported outcomes, these measures are inherently subjective and highly susceptible to placebo effects, external stressors, and the Hawthorne effect. Despite these limitations, the consistent improvement in quality of life across most studies provides preliminary practical support for integrating dietary recommendations into clinical care, even if mechanistic insight remains incomplete.

Diverse Nutritional Strategies

The included studies investigated a diverse range of nutritional strategies, including a pseudoallergen-free diet, a low-histamine diet, and broader anti-inflammatory dietary patterns such as the Mediterranean diet. Several interventions overlapped in their restriction of processed foods, additives, and naturally occurring biogenic amines. These dietary approaches generally have an anti-inflammatory, whole-food focus, limiting processed foods and additives. However, there are key differences. For example, the Mediterranean diet may include moderate amounts of wine and aged cheese [6], whereas these are strictly avoided in low-histamine and pseudoallergen-free diets [8,31-34]. Additionally, certain fruits and vegetables, such as lemons, oranges, pineapples, strawberries, tomatoes, and spinach, are prohibited in low-histamine and pseudoallergen-free diets but encouraged in the Mediterranean diet. The study by Matano et al. found that CSU was associated with egg intake (odds ratio: 1.05) and a higher BMI, whereas patients with uncontrolled CSU demonstrated an increased intake of coffee and caffeine-rich beverages (odds ratio: 1.01) [7]. Egg consumption is discouraged in the pseudoallergen-free diet, yet it is permitted in the Mediterranean and low-histamine diets. Coffee is restricted in both low-histamine and pseudoallergen-free diets. Future comparative analyses should aim to clarify these areas of overlap and divergence, which may help isolate the most relevant dietary triggers for CIU and support more practical, evidence-based guidance for patients. Importantly, given the nutritional adequacy and long-term sustainability of the Mediterranean diet, it may provide a more feasible baseline framework upon which targeted restrictions can be layered for CIU management.

Disease Pathology

CIU is characterized by the absence of a specific external trigger and by symptoms lasting longer than six weeks, distinguishing it from chronic inducible urticaria and acute urticaria, respectively [1,2]. Therefore, the goal of this paper was not to identify triggers but rather to explore the existing literature to determine what role, if any, diet may play in CIU management. As discussed in the background, the current understanding of CIU pathogenesis includes inappropriate mast cell activation through IgE- or IgG-autoantibody-mediated mechanisms and diverse other pathways [12-15]. Despite advances in our understanding of CIU pathophysiology, controversies surrounding the role of diet persist [15]. Our findings demonstrate that very little recent literature on this topic has passed critical appraisal, with only eight of the 121 initially identified articles meeting the final stage of analysis. Of these eight studies, only four [8,31,34,35] examined biological or inflammatory markers, highlighting the need for further mechanistic research into how dietary interventions may influence immune pathways in CIU.

Beyond inflammatory marker research, emerging areas of investigation include the gut microbiome, intestinal permeability, probiotics, omega-3 fatty acids, and other nutritional approaches that may influence immune regulation and mast cell activity. These topics may further clarify the relationship between diet and CIU but were not represented among the studies meeting our predefined inclusion criteria and therefore remained outside the scope of the present review. Future studies integrating these mechanistic pathways with clinical dietary interventions may provide a more comprehensive understanding of nutritional management in CIU.

Furthermore, six of the eight articles reported improved symptoms or favorable associations with differing dietary factors or interventions. This suggests that dietary modification may have a role in CIU management and may influence CIU activity through alterations in the gut microbiome, neuroimmune interactions, or other immunomodulatory pathways. We hypothesize that dietary modification may lower the body’s inflammatory threshold, thereby contributing to improved disease control.

The Research Question

The guiding research question is: “Does diet play a role in the management of CIU in individuals aged 17 years or older, and what evidence exists to support it?” A review of the literature revealed that six of the eight final articles reported measurable benefits or favorable associations between dietary factors or modifications and CIU disease activity. Of the initial 121 articles identified, only eight met the criteria through primary and secondary screening and critical appraisal. Beyond clinical outcomes, these studies also evaluated quality of life, suggesting that, for individuals aged 17 years or older with CIU, potential improvements in well-being may outweigh the nutritional restrictions or lifestyle adjustments required by dietary changes.

Limitations

Several limitations were identified across the included studies. Several studies included small sample sizes ranging from 22 to 60 participants, limiting statistical power and reducing the generalizability of the findings. Diversity among study designs, dietary interventions, outcome measures, and statistical reporting limited direct comparisons across studies and prevented a quantitative meta-analysis. Additionally, important confounding factors, such as physical activity and psychological stress, were often not controlled for. In some studies, the use of micronutrient supplements, NSAIDs, or aspirin alongside the dietary interventions was not reported, limiting the ability to isolate the effects of diet alone.

## Conclusions

This review highlights the emerging, albeit limited, evidence supporting a role for dietary modification in the management of CIU/CSU. Of the eight studies that met the inclusion criteria, six reported improvements in disease activity or quality of life or favorable associations between dietary factors and clinical outcomes, suggesting that nutritional strategies, particularly those limiting histamine or pseudoallergens, may offer clinical benefits. However, the current body of literature remains constrained by small sample sizes, short intervention durations, and limited exploration of immunologic mechanisms. Despite these limitations, the reported patient improvements across diverse geographic and dietary contexts underscore the potential relevance of diet as an adjunctive approach to CIU/CSU management. Future research should prioritize larger sample sizes, longer-term trials, and greater control of participant-level confounding variables to clarify how specific dietary components influence CIU/CSU pathophysiology and immunologic regulation. Future studies should also account for sex as a biological variable that may influence interactions among diet, the microbiome, and immune regulation. Until such data are available, clinicians may consider individualized dietary counseling for selected patients as part of a holistic management strategy, balancing potential symptom relief and quality-of-life improvements against the challenges of dietary restriction.
